# Reversible Cysteine Acylation Regulates the Activity of Human Palmitoyl-Protein Thioesterase 1 (PPT1)

**DOI:** 10.1371/journal.pone.0146466

**Published:** 2016-01-05

**Authors:** Michal Segal-Salto, Tamar Sapir, Orly Reiner

**Affiliations:** The Department of Molecular Genetics, Weizmann Institute of Science, 76100 Rehovot, Israel; University of Melbourne, AUSTRALIA

## Abstract

Mutations in the depalmitoylating enzyme gene, *PPT1*, cause the infantile form of Neuronal Ceroid Lipofuscinosis (NCL), an early onset neurodegenerative disease. During recent years there have been different therapeutic attempts including enzyme replacement. Here we show that PPT1 is palmitoylated *in vivo* and is a substrate for two palmitoylating enzymes, DHHC3 and DHHC7. The palmitoylated protein is detected in both cell lysates and medium. The presence of PPT1 with palmitoylated signal peptide in the cell medium suggests that a subset of the protein is secreted by a nonconventional mechanism. Using a mutant form of PPT1, C6S, which was not palmitoylated, we further demonstrate that palmitoylation does not affect intracellular localization but rather that the unpalmitoylated form enhanced the depalmitoylation activity of the protein. The calculated Vmax of the enzyme was significantly affected by the palmitoylation, suggesting that the addition of a palmitate group is reminiscent of adding a noncompetitive inhibitor. Thus, we reveal the existence of a positive feedback loop, where palmitoylation of PPT1 results in decreased activity and subsequent elevation in the amount of palmitoylated proteins. This positive feedback loop is likely to initiate a vicious cycle, which will enhance disease progression. The understanding of this process may facilitate enzyme replacement strategies.

## Introduction

Neuronal Ceroid Lipofuscinosis (NCL) represents a group of common progressive encephalopathies of children, which is further divided to ten different subtypes [1–4]. The pathological hallmarks of NCL are the accumulation of autofluorescent storage material in the brain and other tissues, progressive psychomotor retardation, visual failure, and seizures. Mutations in the palmitoyl protein thioesterase gene (*PPT1)* cause the infantile form of the disease [5], whereas less severe mutations in the same gene result in the juvenile form [6]. The crystal structure of the protein assisted in understanding the variety of phenotypes associated with different mutations [7]. PPT1 (MIM256730, EC 3.1.2.22), is a thioesterase that normally functions to remove long-chain fatty acids from modified cysteine residues in proteins. The process of long-chain fatty acid addition, termed *S-*acylation, is often referred to as palmitoylation, since palmitate is the main lipid found at *S*-acylation sites. This process of post-translational modification is reversible and dynamic, and the transition period between the palmitoylated and depalmitoylated protein forms is estimated to vary between minutes to hours [8, 9]. Palmitate is a 16-carbon saturated fatty acid that is attached to proteins post-translationally. This modification increases protein hydrophobicity, facilitates protein interactions with lipid bilayers, and can profoundly alter protein sorting and function. Whereas other attachments of fatty acids, such as myristoylation and isoprenylation, are stable and permanent modifications, the thioester bond that links protein to palmitate is labile and thus reversible [10]. Thus, *S*-acylation is conceptually reminiscent of other signaling induced modifications, such as phosphorylation, ubiquitination, and acetylation, although the current knowledge about *S*-acylation and its cellular effects is much more limited. One reason for the relatively slow progress in the field is the lag in the development of techniques enabling relatively rapid and safe means to monitor palmitoylation [11, 12]. *S*-acylation has been shown to play an important role in the regulation of protein trafficking, in particular in the nervous system [13] (for further information see reviews [10, 14–18]). Twenty-three palmitoylation enzymes with a conserved DHHC motif [19] have thus far been discovered in mammals, while the known repertoire of depalmitoylation enzymes is rather limited.

PPT1, the best-studied depalmitoylation enzyme, is a soluble lysosomal protein, and it has also important roles in other cellular localizations [20, 21] (review [22]). The protein is also found in the cell media, and its secretion is mediated by the presence of a consensus signal sequence [20]. As many other soluble lysosomal enzymes, PPT1 was found to be targeted to lysosomes through the mannose 6-phosphate receptor pathway [20, 21, 23–25]. Mannose 6-phosphate receptors bind their cargoes either in the trans Golgi network or they can bind extracellular cargos on the cell surface prior to being endocytosed. The glycosylation of PPT1 is of importance for proper processing through this pathway. Thus, PPT1 may be detected in organelles and vesicles of the secretory pathway, as well as endocytosed vesicles [26]. PPT1 function in the Endoplasmic Reticulum (ER) is also evident, as part of the pathophysiology of NCL which involves malfunctioning ER [27, 28]. In neurons, PPT1 was detected in axons, synaptic vesicles and synaptosomes. These localizations are of importance for the understanding of the disease mechanism since in the knockout mouse model the recycling of synaptic vesicles was affected [29–34]. During recent years several therapeutic interventions have been attempted (review [35, 36], these included enzyme replacement [37–39], small molecule treatments [40–43], gene-therapy combined with others [44, 45]. Here we show that PPT1, an important depalmitoylation enzyme, is regulated through reversible *S*-acylation. The main PPT1 palmitoylation site is on cysteine six within the signal peptide, and the palmitoylated protein is detected in the cell lysates and in the medium. The enzymatic activity of the nonpalmitoylated PPT1, C6S, mutant protein is higher than that of the wildtype, which was attributed to an increase in Vmax. Our results suggest that the addition of a palmitate group is reminiscent of adding a noncompetitive inhibitor. Thus, we reveal the existence of a positive feedback loop, where palmitoylation of PPT1 results in decreased activity and subsequent elevation in the amount of palmitoylated proteins. The understanding of this process may affect our understanding of the pathophysiology of NCL and may assist the design of better therapeutic agents.

## Materials and Methods

### Plasmids

Human PPT1 was cloned from pCMV::PPT1 received from Dr. Sandra Hofmann (University of Texas, Southwestern Medical Center, Dallas) digested by EcoRI and SalI and cloned into pCAGGS digested with EcoRI and XhoI. Site directed mutagenesis of PPT1 enzyme, *PPT1* C6S mutation, was generated using the following primer:

5’ GGAATTCATGGCGTCGCCCGGCAGCCTG 3’

The mouse *Ppt1* cDNA was obtained from the Riken Mouse Encyclopedia Archive positioned in the Weizmann Institute of Science, and was subcloned into pRSET-A (Invitrogen, Carisbad, CA) to generate a six-histidine tagged protein using PCR primers. pEF-BOS-Ha-DHHC plasmids were received from Dr. Masaki Fukata and described previously [19]. The following fluorescent protein expression constructs were received from Dr. Michael W. Davidson from the National High Magnetic Field Laboratory, Florida State University, Tallahassee, Florida: the Golgi marker plasmid mCherry-Golgi (GalT)-7 that expresses 82 amino acids of β 1,4-Galctosyltransferase and mCherry, the Endoplasmic Reticulum marker, Ds-Red2- Endoplasmic reticulum 5 that encodes the calreticulin signal sequence, and the Ds-Red protein with the KDEL sequence at its C-terminus, and the lysosomal marker mApple-Lysosome-20, which encodes for Lamp1 (lgp120) (407 amino acid) in fusion with mApple.

### Antibodies

Anti-PPT1 antibodies were raised by injecting the six-histidine tagged mouse PPT1 recombinant protein purified from bacteria into two rabbits. The antibodies were used for immunoprecipitation, western blot and immunohistochemistry. Information regarding the verification of the antibodies can be found in S1 Fig. Endogenous human PPT1 from hES cells were immunoprecipitated and blotted using mouse anti-human PPT1 (Origene, TA800501). Mouse monoclonal anti-HA antibodies (Convance, Berkeley, California), were used for western blot analysis a 1:1000 dilution. Secondary antibodies: for staining Alexa Fluor® 488 goat anti-rabbit IgG (H+L) was purchased from Molecular Probes (Invitrogen, Carisbad, CA). For Western blots Peroxidase-conjugated AffinitiPure Goat Anti-Rabbit IgG (H+L) and Peroxidase-conjugated AffinitiPure Goat Anti-Mouse IgG F(ab’)2 were purchased from Jackson ImmunoResearch Laboratories Inc. (West Grove, PA, 19390).

### Cell culture and transfections

Human HEK293 and Green monkey, COS7 cells were grown in DMEM medium supplemented with 10% fetal bovine serum (Biological Industries, Kibbutz Beit Haemek, Israel), 100 U/ml penicillin and 0.1mg/ml streptomycin (Biological Industries, Kibbutz Beit Haemek, Israel) at 37°C 5% CO_2_. The media for growing human embryonic stem cells were previously described [46]. Human embryonic stem cells (WIBR3, NIHhESC-10-0079) [47] were mutated using the RNA-guided CRISPR–associated nuclease Cas9 as an effective mean to introducing targeted loss-of-function mutations [48, 49] at the *PPT1* locus. The usage of the human ES cells was approved by the Weizmann IRB committee. The guide RNA was designed using http://crispr.genome-engineering.org/ and cloned into pX330, the primers used were 5’-caccGTTGGACTCCCTCGATGCCC and 5’-aaacGGGCATCGAGGGAGTCCAAC, methods were taken from [50–52]. *PPT1* knockout was verified by DNA sequence, real-time qPCR, and Western blot shown in S1 Fig.

### Immunostaining

COS7 cells were platted on 13-mm thick cover slips (Menzel-Glaser, Braunschweig, Germany). Twenty-four hours after transfection, cells were fixed for 20 minutes at room temperature in PHEM buffer (60 mM PIPES-KOH, pH 6.9, 25mM HEPES, 10mM EGTA. 1 mM MgCl_2_) with 4% PFA and 0.08% glutaraldehyde in PBSx1, washed 3 times in PBS and permeabilized using 0.1% Triton X-100 for 25 minutes at room temperature. After quenching two times with 0.25% NH_4_Cl in PBS for 10 min, the cells were subjected to blocking for 15 minutes in PBS supplemented with 0.1% BSA (Sigma, Rehovot, Israel), cover slips were incubated with the indicated antibodies for 1 hour at 37°C, in the blocking solution. Following several washes, secondary antibodies were applied for 1h in the dark, at 37°C, in blocking solution. Finally, cover slips were washed, incubated with 4',6-diamidino-2-phenylindole (DAPI) for 10 min and mounted with Vectashield (Vector, CA, USA) and visualized using wide-field microscopy (DeltaVision, Applied Precision, CA, USA). Images were processed using the DeltaVision system package and Imaris software (for 3D images) (Bitplane, Saint Paul, USA).

### Immunoblots and Immunoprecipitation

Adherent cultured cells were washed in cold PBS and collected by cell scrapers. The cells were collected by low speed centrifugation. Cell pellets were either flash frozen in liquid N_2_ and stored at -80°C for later lysis or were lyzed directly by resuspension in lysis buffer containing 150 mM NaCl, 50 mM Tris-HCl (pH 7.4), 5 mM EDTA, 1% Triton X-100 supplemented with a Protease Inhibitors Cocktail (Sigma, Rehovot, Israel). For immunoblots, 10–20 microgram of proteins were mixed with SDS sample buffer and separated by SDS-PAGE. The rest of the protein lysates were incubated with appropriate antibodies for 2 hours at 4°C. Following this, 15 μl (bed volume) of protein A/G agarose (Santa Cruz, San Diego, CA) pre-blocked in lysis buffer supplemented with 10 mg/ml BSA (Sigma, Rehovot, Israel), was added to each sample for additional 1 hour. Immunoprecipitated proteins were precipitated by centrifugation and washed 3 times in lysis buffer. The proteins were eluted from the beads by the addition of SDS sample buffer, boiled for 3 minutes, separated on SDS-PAGE and subjected to western blot analysis with the indicated antibodies.

### Metabolic labeling, Click Chemistry

Transfected HEK293 cell lines were grown as described above. The metabolic labeling and Click chemistry were performed as previously described [12, 53]. Briefly, 17-octadecynoic acid (17-ODYA) (BioMol, Plymouth Meeting, PA, USA) was dissolved in DMSO to make a 25 mM (1000x) stock, it was diluted in media and briefly sonicated, then added to a confluent 10 cm plate of cells. Cells were labeled overnight. For half-life determination, cells were washed twice with fresh media, and then fresh media supplemented with 200μM palmitic acid (Sigma, Rehovot, Israel) was added for the indicated time points (1, 2, and 3 hours). Following metabolic labeling, cells were harvested, washed once with ice-cold phosphate buffered saline (PBS) and pelleted at 1000g for 5 min. Cells were directly lysed in IP buffer; 0.2 ml 50 mM Tris-HCl pH 7.4, 150 mM NaCl, 5mM EDTA, 1% Triton X-100 (Sigma, Rehovot, Israel) plus protease inhibitors and then incubated for 30 minutes on ice. Cell lysates were collected after centrifuging at 15000g for 15 min at 4°C to remove cell debris. A sample was taken for lysate control. The inspected proteins were immunoprecipitated as described above using the appropriate antibodies. The immunoprecipitated proteins were reacted with 40 microliter freshly premixed click chemistry reaction cocktail Alexa Flour® 647 azide or Tetramethylrhodamine azide (Invitrogen, Carlsbad, CA, USA) (100 micromolar, 10 mM stock solution in DMSO), tris(2-carboxyethyl) phosphine hydrochloride (TCEP) (1 mM, 50 mM freshly prepared stock solution in deionized water), tris[(1-benzyl-1H-1,2,3-triazol-4-yl)methyl methyl] amine (TBTA) (100uM, 1.7 mM stock solution in DMSO) and CuSO4 ·5H2O (1 mM, 50 mM freshly prepared stock solution in deionized water) in PBS for 1 hr at room temperature. The reactions were terminated by the addition of 10 microliter5× reducing SDS-loading buffer with low concentration of DTT (40% glycerol, 200 mM Tris-HCl pH 6.8, 8% SDS, 0.4% bromophenol blue, 20 mM DTT) and heated for 5 min at 95°C; samples were loaded and separated by SDS-PAGE (8%, 10% or 12% PAGE gel). Gels were scanned using an Amersham Biosciences Typhoon 9400 variable mode imager (excitation 633 nm, emission 670 nm filter) and then gels were immunoblotted and analyzed with specific antibodies.

### Hydroxylamine treatment of samples

Hydroxylamine treatment was done by addition of 1M freshly made hydroxylamine solution in the SDS-loading buffer. Hydroxylamine was prepared as a 10M stock solution in PBSx1 and the pH was adjusted to 7.4 using HCl.

### PPT1 enzymatic activity assay

COS7 cells were transfected and grown for additional 24 hours. Cells were collected and sonicated in double distilled water supplemented with protease inhibitors. The cell lysates (0.1 μg) or the cell media (1μl) were added to the reaction buffer, similar to previously described [54], which was composed of 4-methylumbelliferyl-6-thiopalmitoyl-β glucoside in Pi/Ci buffer (double concentrated McIlvains phosphate/citric-acid buffer, 0.4M Na_2_HPO_4_/0.2M Citric-acid, pH 4, and 0.00625% Triton X-100). The control reaction, which estimated the spontaneous decomposition of the substrate, included 0.2% BSA. The reactions were incubated for 1 hour at 37°C and stopped by the addition of carbonate buffer pH = 10.7 with 0.025% Triton X-100. The fluorescence of 4-methylumbelliferone was measured by Modulus microplate (Turner Biosystems) with a UV filter (excitation 460 nm, emission 510–570 nm). The background readout was subtracted and the values were calculated for nmol/h/mg units. The control or human PPT1 values were normalized to 100% activity.

### Statistical analysis

Statistical analysis and curve fitting were done using Prism 5 for Mac OS X (GraphPad Software, Inc., La Jolla, CA) www.graphpad.com.

## Results

### PPT1 is a Palmitoylated protein

**Fig 1:**
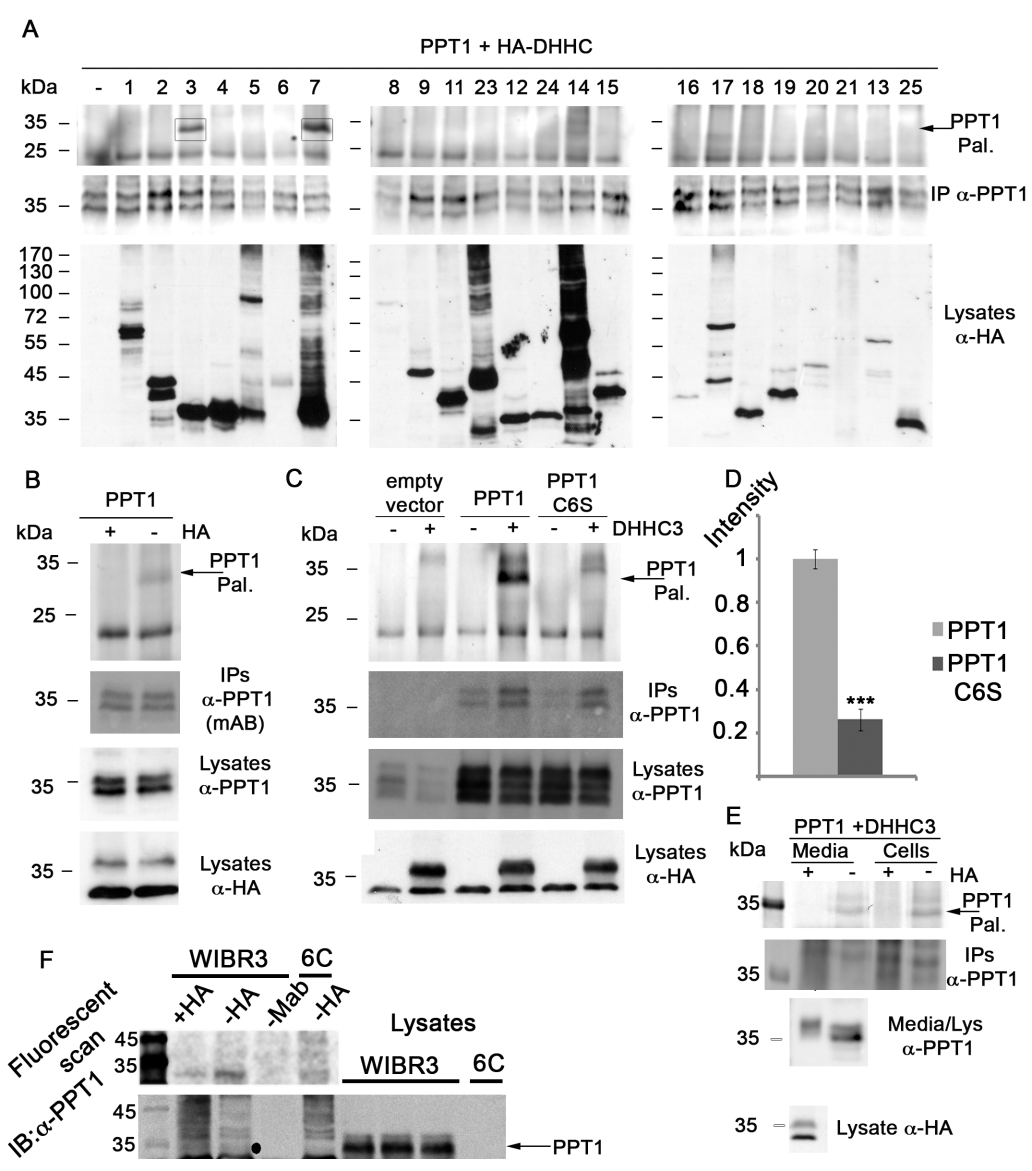
PPT1 is palmitoylated by DHHC3 and DHHC7. A) PPT1 was co-transfected with HA tagged DHHC enzymes to HEK293 cells. The cells were metabolically labeled with 17-ODYA and PPT1 was immunoprecipitated with anti-PPT1 antibodies. A fluorescent tag was introduced by click chemistry and samples were separated by SDS-PAGE. Fluorescent scanning of the gels detected that PPT1 is palmitoylated by DHHD3 and DHHC7 (top panel, boxed). The relative amount of immunoprecipitated PPT1 is shown (middle panel). The expression of the DHHC enzymes in the cell lysates was verified by immunoblotting with anti-HA antibodies (low panel). B) PPT1 was co-transfected with HA-DHHC3 followed by metabolic labeling, immunoprecipitation of PPT1 and click chemistry. One reaction was treated with hydroxylamine and one reaction with PBS. The addition of hydroxylamine eliminated the fluorescent signal of PPT1 (upper panel) demonstrating that the signal is specific for palmitoylation. Similar amounts of PPT1 were immunoprecipitated and expressed (middle panels). The level of HA-DHHC3 expression in the cell lysates was detected by anti-HA antibodies (lower panel). C) PPT1 is palmitoylated on cysteine residue 6 and can be detected in the cell media. Empty vector, PPT1 or PPT1 C6S were co-expressed with HA-DHHC3 in HEK293, followed by metabolic labeling with 17-ODYA, immunoprecipitation and click chemistry. The control treatment results in a low background fluorescent signal probably due to low levels of expression of endogenous PPT1. The expression and amount of immunoprecipitated PPT1 and PPT1 C6S are similar as detected by immunoblotting with anti-PPT1 antibodies (middle panels). The expression of HA-DHHC3 in was detected by immunoblotting with anti-HA antibodies (lower panel). D) The fluorescence signal intensity for PPT1 and PPT1 C6S were quantified and normalized relative to the amount of immunoprecipitated PPT1 using four independent repeats. The fluorescent signal of PPT1 signal is almost five fold higher than that observed with PPT1 C6S. E) Palmitoylated PPT1 can be detected in cells and media and the signal is reduced following hydroxylamine treatment. PPT1 was co-expressed with HA-DHHC3 in HEK293 cells followed by metabolic labeling both cells and media were collected and PPT1 was immunoprecipitated and labeled using click chemistry. The fluorescent signal of PPT1 palmitoylation is shown in the upper panel. This signal is eliminated by the addition of hydroxylamine. The amount of immunoprecipitated PPT1 and the level of expression in the lysates are shown in the middle panels and in both cell and media by immunoblotting with anti-PPT1 antibodies. The expression of HA-DHHC3 was detected by immunoblotting with anti-HA antibodies in the lower panel. E) Palmitoylation of endogenous PPT1 in human embryonic stem cells. WIBR3 wildtype and 6C, PPT1 knockout clone generated using CRISPR/Cas9 were metabolically labeled with 17-ODYA, followed by immunoprecipitation of PPT1 and click chemistry. We could distinguish fluorescent band appeared in the–HA treatment, that band could be eliminated by hydroxylamine (+HA). The band did not appear in negative control of immunoprecipitation with beads only (-mAB) or in the immunoprecipitation from the PPT1 knockout 6C clone. The lower panel confirms that PPT1 was immunoprecipitated using Western blot.

PPT1 is a depalmitoylation enzyme, and while testing several putative substrates of PPT1, we noticed that PPT1 itself is a palmitoylated protein. To directly test this possibility, PPT1 was individually transfected to HEK293 cells in combination with expression constructs of each one of the 23 known palmitoylating enzymes (also dubbed palmitoyltransferases, PATs or DHHCs). The capability of these enzymes to palmitoylate PPT1 was then assayed by metabolic labeling of HEK293 cells with 17-ODYA (an analog of palmitate) (Fig 1A) [12, 53]. Cell lysates were subsequently subjected to click chemistry, which allowed tagging of the metabolically labeled molecules (with 17-ODYA) with a fluorescent tag. Significantly, fluorescent scanning of the gels revealed tagging of PPT1 in the presence of two palmitoylating enzymes, DHHC3 and DHHC7 (Fig 1A upper panel, boxes). Similar experiments were conducted with mouse PPT1, which is also palmitoylated by DHHC3 and DHHC7 (S2 Fig). The results obtained with DHHC21 are not conclusive since its expression was not detectable by Western blot analysis (Fig 1A and S2 Fig). Nevertheless, since this enzyme was active on the mouse protein, we do believe it was expressed also in case of the human PPT1 experiment. The mouse protein contains additional cysteine residues and additional enzymes palmitoylated it, since this study was focused on the human protein, we did not continue to investigate the palmitoylation of the mouse PPT1 protein.

Myristoylation is an irreversible protein modification in which a myristoyl group (derived from myristic acid) is covalently attached via an amide bond to the alpha-amino group of an N-terminal amino acid of a nascent polypeptide. Hydroxylamine treatment removes S-palmitoylation modifications but does not remove N-palmitoylation or myristoylation. Therefore, to confirm that PPT1 is S-palmitoylated and not N-palmitoylated or myristoylated, we added neutral hydroxylamine. Indeed, in the presence of DHHC3, the fluorescent signal was practically eliminated following the addition of hydroxylamine, indicating that PPT1 was palmitoylated by this enzyme (Fig 1B top panel).

### Cysteine 6 of PPT1 is palmitoylated

We next investigated which of the eight cysteine residues present in the human PPT1 protein undergoes palmitoylation. Using the prediction program, CSS-Palm version 4, the 6^th^ cysteine residue in PPT1 received the highest score for a tentative palmitoylation site [55]. We then tested whether the C6S mutant PPT1 can still undergo palmitoylation in the presence of DHHC3 (Fig 1C). In the absence of PPT1, some background fluorescence was detected, probably due to low expression levels of the endogenous PPT1 protein, which can be detected in the lysates blotted with anti-PPT1 antibodies. This low level of expression was not sufficient to detect the protein in the IP blot. However, when the PPT1 C6S mutant protein was tested, the fluorescent signal decreased by almost five fold relative to the wild type protein (Fig 1C top panel and 1D for the intensity of the fluorescence). We confirmed that the decrease in the intensity of the palmitoylated band was not a consequence of variations in either the levels of the substrates (PPT1 or PPT1 C6S) or of the enzyme (DHHC3) (Fig 1C lower panels). Nevertheless, the existence of some background fluorescence may suggest the presence of an additional, less prominent site, which undergoes palmitoylation. Since the residual signal was low, we did not pursue the characterization of a putative second site for palmitoylation further. These results suggest that cysteine 6 is the main palmitoylated site in PPT1.

The palmitoylation site, C6, is located within the conventional signal sequence of PPT1 at the N-terminus of the protein. It was therefore unexpected to detect a fluorescent signal corresponding to palmitoylated PPT1 in the cell medium (Fig 1E). Palmitoylation was performed by DHHC3 whose expression was detected by western blot analysis (Fig 1E lower panel). Consistently, hydroxylamine reduced the thioester bond between the cysteine and the 17-ODYA, and eliminated the fluorescent signals in the extra- and intra- cellular samples (Fig 1E), indicating that the observed signal in both cases was palmitoylation. Since the signal peptides are usually cleaved following secretion, these results suggest that a subset of the PPT1 proteins may undergo secretion through an unconventional pathway, which allows the secreted protein to retain an intact signal sequence. Western blot analysis of PPT1 in the cell lysates and the medium detected a mobility shift, which is consistent with previously detected difference in the glycosylation patterns of intracellular and extracellular PPT1 [21].

Next we tested whether PPT1 is palmitoylated *in vivo*. We generated a PPT1 null control cell line using CRISPR/Cas9 gene editing technology. Following metabolic labeling, immunoprecipitation and click chemistry a fluorescent signal was evident only in the wild-type human embryonic stem cells (WIBR3, NIHhESC-10-0079) but not in PPT1 deficient cells and not in cells treated with hydroxylamine (Fig 1F).

Collectively, our results indicate that PPT1 is a palmitoylated protein *in vivo*. It undergoes palmitoylation on C6, which is located in the signal peptide of the protein. Palmitoylated PPT1 was detected both in the cell lysates and in the cell media. Furthermore, our results may suggest that a fraction of the PPT1 proteins present within the cell, undergoes secretion through an unconventional pathway and retains the signal sequence.

**Fig 2:**
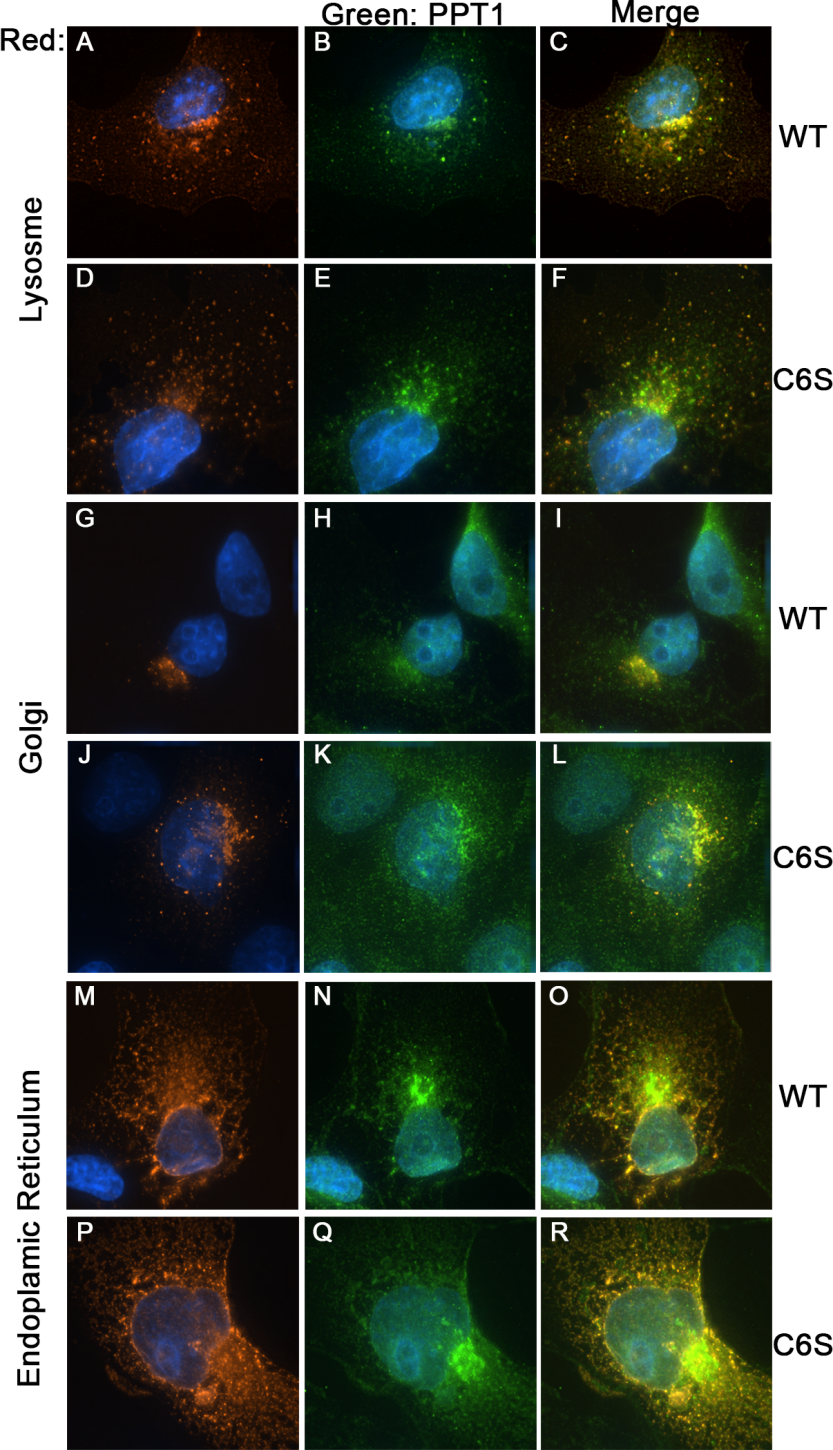
PPT1 palmitoylation does not affect its subcellular localizations. The localization of PPT1 and PPT1 C6S was examined by co-transfection with fluorescently tagged cellular markers. A-F) Colocalization of PPT1 (A-C) and PPT1 C6S (D-F) to the lysosome marker Lamp1. Both PPT1 and PPT1 C6S are partially localized to the lysosome compartment in COS7 cells. G-L) PPT1 and PPT1 C6S localized also to the Golgi as demonstrated by Golgi marker coexpresssion. M-R) Colocalization of PPT1 and PPT1 C6S to the endoplasmic reticulum is demonstrated using a fluorescent tag attached to the KDEL ER retention signal. The experiments were repeated three times and in each experiment at least 25 cells were imaged.

Next, we explored possible effects of PPT1 palmitoylation on the localization and function of the protein. The intracellular localization of transfected C6S PPT1 was compared to that of the WT form of PPT1 using a battery of fluorescent intracellular organelles expression plasmids. Our analysis included organelles to which PPT1 is known to be localized to; lysosomes, the Golgi apparatus, and the ER (Fig 2). Partial colocalization of PPT1 with lysosomes was noted both with the wild type protein (Fig 2A–2C) and the C6S mutated form (Fig 2D–2F). The wild type (Fig 2G–2I),(Fig 2M–2O) and the mutant (Fig 2J–2L),(Fig 2P–2R) PPT1 proteins were also detected in the Golgi and the ER, respectively. The Pearson correlation coefficient was analyzed in the images. There was no statistically significant difference in the coefficients obtained in the colocalization of either the wild type or the mutant PPT1 with the different subcellular markers. Therefore, we conclude that PPT1 palmitoylation does not grossly affect the intracellular localization of the protein.

### PPT1 palmitoylation and its depalmitoylation activity

**Fig 3:**
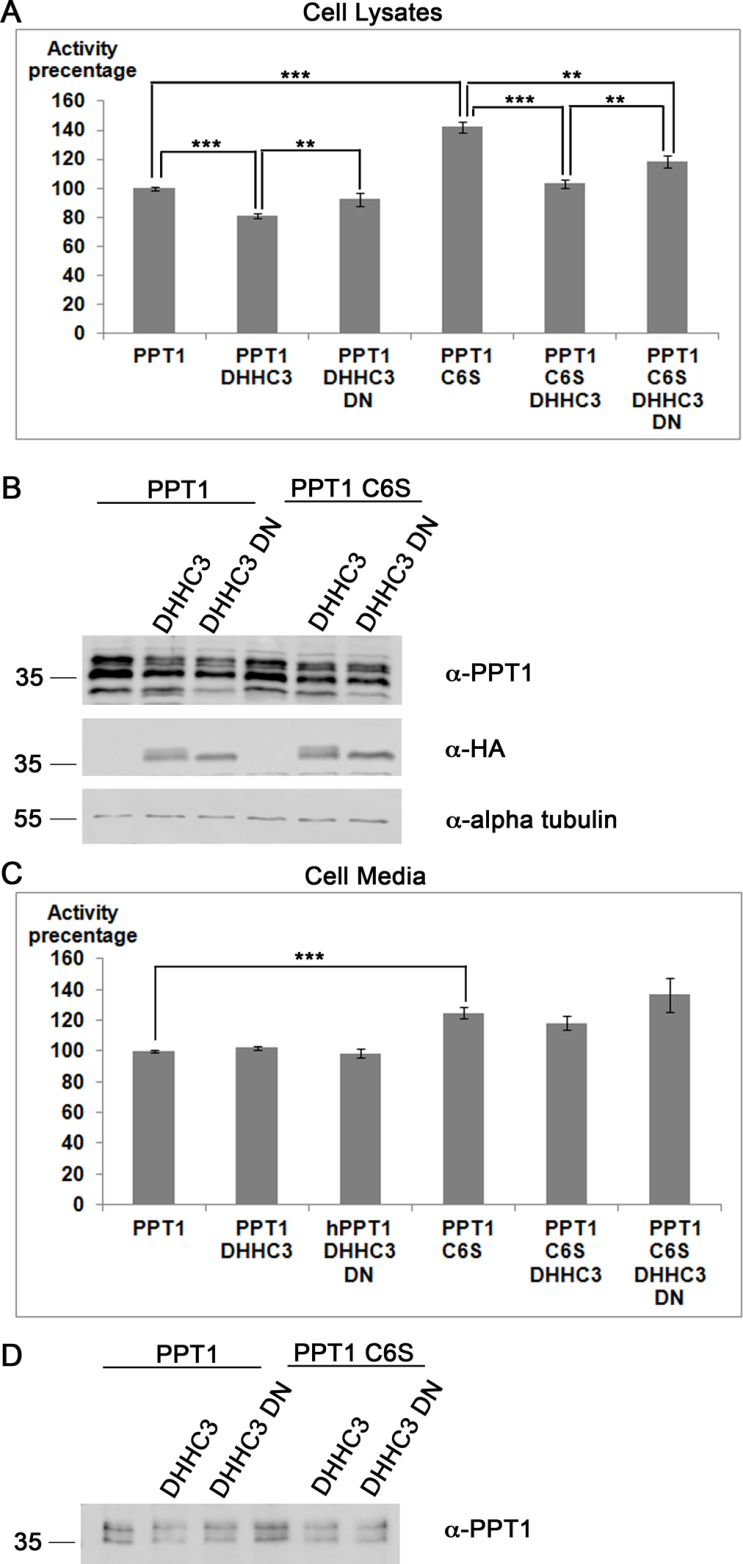
PPT1 C6S exhibits elevated enzymatic activity in the cell lysates (A) and the cell media (C). The activity of PPT1 in COS7 cells was monitored following the overexpression of either PPT1 or PPT1 C6S, or with the addition of either HA-DHHC3 or HA-DHHC3 dominant negative mutant. The activity of PPT1 was defined as 100%, and the activity in other treatments were calculated accordingly (N = 3). A) In the cell lysates, the addition of HA-DHHC3 reduced PPT1 activity to 81%, while the addition of the dominant negative HA-DHHC3 resulted in 92% activity. The expression of the PPT1 C6S mutant resulted in 142% activity, whereas the co-expression with HA-DHHC3 resulted in activity levels similar to that of the wildtype enzyme (103%). The addition of the HA-DHHC3 dominant negative enzyme elevated the activity to 118%. B) Western blots showing the relative expression of PPT1, HA-DHHC3, and alpha-tubulin (loading control). C) In the cell media the activity of either the single transfected PPT1 or PPT1 C6S differed significantly. However, this effect was eliminated following the co-expression with either active DHHC3 or with its dominant negative mutant. D) The expression of PPT1 in the media was examined by western blot analysis. In the presence of DHHC3 wildtype or dominant negative mutant there was a decrease in the amount of PPT1 in the media. **, p<0.01; ***, p<0.001.

We then examined the possibility that palmitoylation may affect the enzymatic activity of PPT1. Lysates from cells transfected with PPT1 wildtype and C6S activity was assayed in the presence of either an active or a dominant negative form of the palmitoylation enzyme, DHHC3 [56]. The release of a fluorescent signal derived from the cleavage of an artificial palmitoylated peptide, 4-MU, which correlates with depalmitoylation activity [54] was measured. The enzymatic activity of PPT1 or of PPT1 C6S in the various treatments was normalized to the activity of individually transfected wild type PPT1 and to the levels of PPT1 protein detected by western blot analysis. Strikingly, when PPT1 was transfected alone, the activity of the nonpalmitoylatable mutant, significantly surpassed that of the wild type enzyme by more than 42±4.1% in the cell lysate and 25±3.7% in the cell medium (Fig 3A,C one way ANOVA, with Tukey's Multiple Comparison Test, n = 9, p<0.001). It should be noted that in the presence of the active and dominant-negative palmitoylation enzymes (DHHC3 and DHHC3 DN), the amount of the secreted PPT1 proteins decreased (see representative western blot Fig 3D). In the cell media, we noticed that in all the combinations, the depalmitoylation activity of PPT1 C6S was higher than that of the wild type protein. In the cell lysates, we also noted that the activity of PPT1 C6S was higher than that of the wild type protein. However, the addition of the active palmitoylating enzyme, DHHC3, resulted in a significant decrease of enzymatic activity both in case of the wild protein and in case of the C6S mutant form (Fig 3A, 18.9±1.3%, 39.0±3.6%, respectively, one way ANOVA, with Tukey's Multiple Comparison Test, n = 9, p<0.001). Importantly, when wild-type PPT1 was cotransfected with the inactive enzyme, DHHC3 DN, the activity of PPT1 was elevated in comparison with the active palmitoylation enzyme, and did not differ significantly from the activity of PPT1 on its own. When PPT1 C6S was expressed in the presence of DHHC3 DN the activity was improved in comparison with the activity noted in the presence of the active palmitoylation enzyme, but still was 23.4±4.3% lower than that observed when PPT1 was expressed individually (Fig 3A, one way ANOVA, with Tukey's Multiple Comparison Test, n = 9, p<0.01). Therefore, we concluded that the mutant PPT1 protein, which exhibited reduced palmitoylation, exhibits higher enzymatic activity in comparison with the wild type protein. In addition, palmitoylation of wild-type PPT1 and also possible palmitoylation of PPT1 C6S mutant proteins resulted in reduced depalmitoylation activity. In case of the PPT1 C6S mutant protein, an additional possibility exists, which is that the effect of DHHC3 on the activity of PPT1 C6S is indirect, and involves other protein interactions.

**Fig 4:**
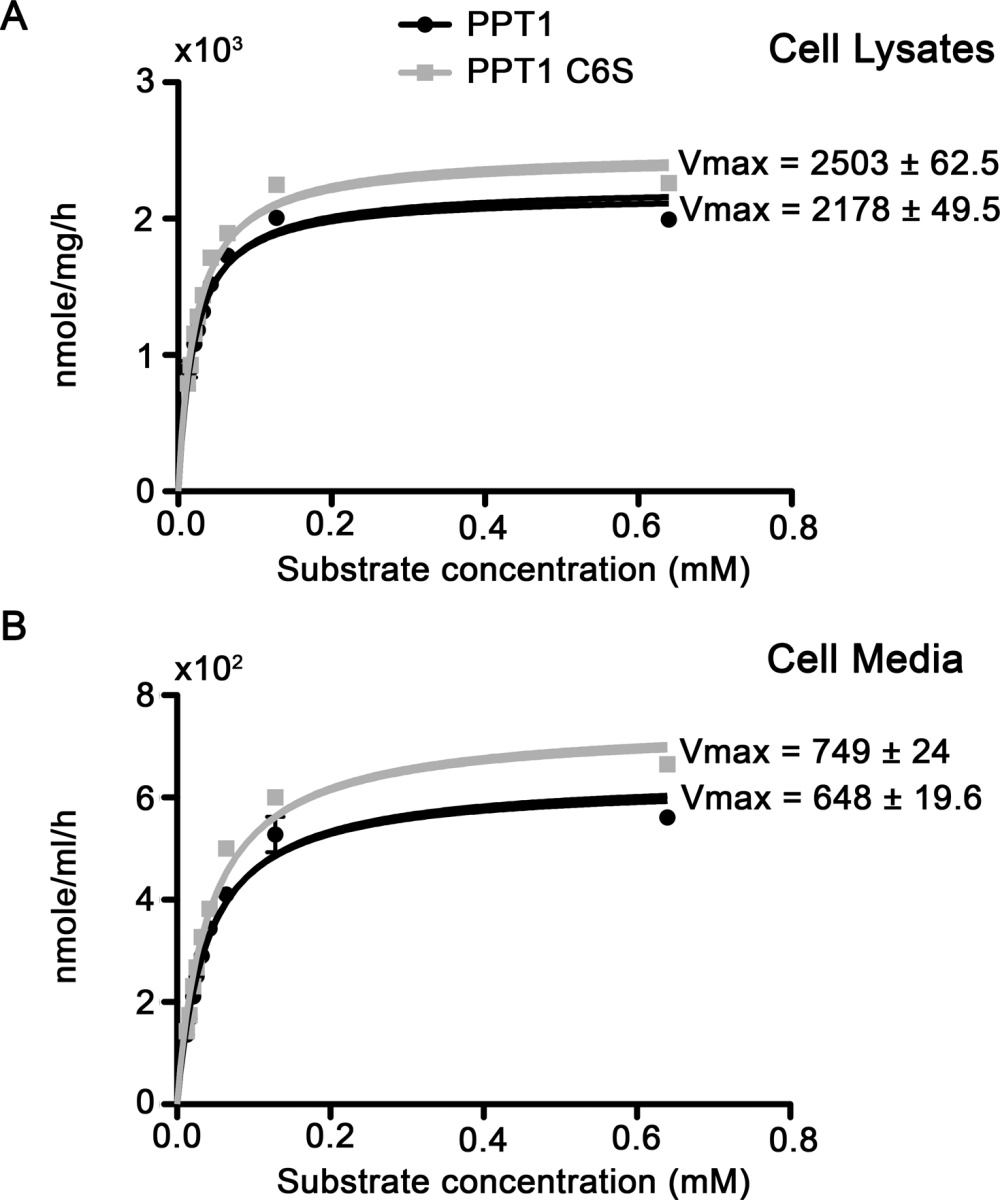
Michaelis-Menten graph of PPT1 and PPT1 C6S. The Km and Vmax of PPT1 and PPT1 C6S were calculated using the Michaelis-Menten equation following plotting the results of enzymatic activity obtained with different concentrations of the substrate (N = 9) A) Michaelis-Menten graph derived from the cell lysates. B) Michaelis-Menten graph derived from the cell media.

We next tested whether palmitoylation affected the affinity in which the enzyme binds to the substrate (Km) and/or the rate of the reaction (Vmax). To this end, cells were transfected with either the wild type enzyme or the non-palmitoylatable mutant protein, and the enzymatic activity was assayed in the cell lysate and medium using increasing concentrations of the substrate. The kinetic data in the cell lysate was analyzed using Prism program, which determined a Vmax of 2178±49.5 nmole/mg/hr for the wild type enzyme and 2503±62.5 nmole/mg/hr for the C6S mutant protein (Fig 4A). The two values differed significantly (p<0.0001), and the differences are evident in the Michaelis-Menten plot (Fig 4A). The Km values were calculated using the Prism software, and did not differ in a statistically significant manner. The Km values were 0.0199±0.00146 and 0.0237±0.0018 mM for the wild type and mutated enzyme, respectively. A similar trend was noted when the kinetic parameters were analyzed in the cell medium; the Vmax for the wild type PPT1 was 628±19.6 nmole/ml/hr, and significantly higher for the C6S mutant, 749±24 nmole/ml/hr (p<0.0001) (Fig 4B). The Km values did not differ significantly for wild type PPT1 and PPT1 C6S (0.0378±0.0031 and 0.0423±0.0035, respectively). We conclude that palmitoylation regulates PPT1 activity in an allosteric fashion, and that the addition of palmitate is conceptually similar to the use of a non-competitive inhibitor.

## Discussion

### PPT1 palmitoylation in the cell and in the media

Our findings revealed that PPT1 can be palmitoylated by DHHC3 and DHHC7. Among all the palmitoylation enzymes, DHHC3 and the closely related enzyme, DHHC7, exhibit the broadest substrate specificity [18, 57]. It is plausible that PPT1 palmitoylation occurs in multiple tissues, since the two palmitoylation enzymes [9, 58], and their substrate, PPT1, are widely expressed [59]. The palmitoylation of PPT1 is likely to occur in association with the Golgi apparatus, since a subset of PPT1 was detected colocalized with the Golgi, which is similar to the reported intracellular localization of endogenous DHHC3 [60, 61] and transfected DHHC7 [58, 62].

Our data suggest that the main PPT1 S-palmitoylation site is cysteine 6 within the signal peptide. Traditionally secreted proteins are N-palmitoylated not S-palmitoylated, however the elimination of the signal following hydroxylamine treatment does not support the possibility that PPT1 is N-palmitoylated.

Furthermore, we detected palmitoylated PPT1 in the cell media, implicating that at least part of PPT1 is secreted in a non-conventional pathway. Alternatively, secreted PPT1 may be palmitoylated in a position different from cysteine 6. However, our observations detected increased enzymatic activity of secreted PPT1 C6S in comparison with the wildtype PPT1. This observation implies that the cysteine residue in position 6 is present in the enzyme post secretion and that the signal peptide is intact. We therefore favor the possibility of unconventional secretion of PPT1, which may occur even if this cysteine is not palmitoylated in the media. Eukaryotic protein secretion normally routes through the ER and Golgi, ending up in secretory vesicles that fuse to the cell membrane. Secretion of proteins without a signal peptide has been initially documented for Plasminogen activator inhibitor-2 (PAI-2), the cytokine interleukin-1 beta [63, 64], and some other proteins (review [65]). As examples were accumulated, an algorithm was composed, which can identify secreted proteins without a signal peptide [66]. Secretion of these proteins does not seem to occur by a single pathway, as diverse distinct mechanisms appear to underlie the release of these proteins. The lack of a unified transport concept led to collectively terming these processes as non-classical protein secretion/export, thus enabling to distinguish them from the classical ER/Golgi-mediated pathway. To the best of our knowledge, PPT1 is a unique example of a protein that is secreted both in the classical and non-classical secretory pathway.

### Palmitoylation and inhibition of enzymatic activity

Our results indicated that palmitoylation reduced PPT1 enzymatic activity. Reduction of enzymatic activity has been previously demonstrated for two mitochondrial enzymes, Methylmalonate semialdehyde dehydrogenase (MMSDH) [67] and carbamoyl-phosphate synthetase 1 (CPS 1) [68, 69], both of which were palmitoylated within their active sites. In contrast, the palmitoylation site of PPT1 is not located within the active site. Its unique position within the signal peptide, thus, may define three groups of PPT1 proteins that are likely to differ in their activity: palmitoylated PPT1, unpalmitoylated PPT1 and following the removal of the signal peptide, cleaved PPT1. Our analysis demonstrated that the maximal velocity of the enzyme that can be palmitoylated is smaller than the non-palmitoylated enzyme. Mechanistically, addition of palmitate is a mode of allosteric regulation of PPT1, as we noticed a significant change in the maximal velocity but not in the Michaelis Menten constant. It has been proposed that during starvation, the inhibition of a few key metabolic enzymes, such as CPS 1 and MMSDH, could be responsible for the switch in the reduction in amino acid oxidation and urea synthesis activity. The fact that PPT1, which is a depalmitoylating enzyme, is itself regulated by palmitoylation may suggest the existence of a positive feedback loop; when PPT1 is palmitoylated, its enzymatic activity will decrease, thus leading to a higher level of palmitoylated proteins.

### PPT1 palmitoylation and NCL

PPT1-related NCL is a devastating early onset neurodegenerative disease, and as such, is considered a likely candidate for gene therapy [70–72] and for enzyme-related therapeutic approaches [37, 73, 74]. Therefore, understanding how PPT1 is regulated may bear practical values. Mutations that affect residues near the active site and in the hydrophobic core of the enzyme are associated with a severe phenotype, whereas mutations in the binding pocket or at the periphery of the enzyme allow for residual activity and are associated with late-onset disease [22]. Our findings may implicate the rationale of treatment of this later group of patients. Reduction of PPT1 palmitoylation will result in increased enzymatic activity, and possibly in a less progressive disease. These possibilities are likely to be aims of future research projects.

## Supporting Information

S1 Fig Verification of anti-PPT1 antibodies. A-B) Overexpression of PPT1 (A) or a control empty plasmid (B) in NIH3T3 cells stained with our rabbit anti-PPT1 antibodies. PPT1 is expressed as discrete puncta with partial localization to perinuclear areas. C-D) Endogenous immuonostaining for mouse PPT1 in MEFs isolated from wildtype mice (C) or PPT1 KO mice (D). PPT1 localizes in various size puncta with preference to perinuclear regions. E-F) Verification of rabbit anti-PPT1 antibodies by western blot. CRISPR/Cas9 gene editing technology was used to generate cell lines deleted for *Ppt1*. The upper western blot represents similar amount of cell lysates that were loaded using anti α-tubulin antibodies. The lower panel is blotted with anti-PPT1 antibodies. A PPT1 specific band appears in the WT lysates inin close proximity to the 35kDa size marker, which is the expected molecular weight of this protein. Less reactivity isdetected in lysates of clones 36, 20 and 84. No PPT1 immunoreactive proteins were detected in clones 56 and 79. We confirmed those results using an *in vitro* activity assay for PPT1 activity (F). Cells lysates were incubated with an artificial PPT1 substrate and enzymatic activity was measured. The enzymatic activity correlated with the amounts of protein detected by Western blot. The wildtype lysate exhibited high enzymatic activity, clone 36 less than half of the wildtype activity, lysates of clones 20 and 84 exhibited very low activity, and lysates of clones 56 and 79 displayed no PPT1 activity at all. G) Detection of PPT1 in cilia isolated from cortical cultures. Cilia were isolated as described using high calcium buffer. We could detect PPT1 protein (black arrow) only in the preparation made from the wildtype cortical culture. Cilia enrichment was demonstrated using anti-Gli3 antibodies (no reactivity was seen in the general lysates).
(TIF)

S2 Fig A) GFP-mouse PPT1 is palmitoylated mainly by DHHC3 and DHHC7 palmitoylation enzymes. GFP-mouse PPT1 was co-transfected with HA tagged DHHC enzymes to HEK293 cells. The cells were metabolically labeled with 17-ODYA and GFP-mouse PPT1 was immunoprecipitated with anti-GFP antibodies. A fluorescent tag was introduced by click chemistry and samples were separated by SDS-PAGE. Fluorescent scanning of the gels detected that PPT1 is palmitoylated mainly by DHHD3 and DHHC7 (top panel), a reduced fluorescent signal was detected following the transfections of DHHC14, 15 and 21. The relative amount of immunoprecipitated PPT1 is shown (middle panel). The expression of the DHHC enzymes in the cell lysates was verified by immunoblotting with anti-HA antibodies (low panel). B) Mouse PPT1 is palmitoylated mainly by DHHC3 and DHHC7. A mouse PPT1 expression construct without any added epitope tag was co-transfected with 23 DHHC enzymes to HEK293 cells. The cells were metabolically labeled with 17-ODYA and immunoprecipitated rabbit polyclonal anti-PPT1 antibodies. A fluorescent tag was introduced by click chemistry and samples were separated by SDS-PAGE. Fluorescent scanning of the gels detected that PPT1 is palmitoylated mainly by DHHD3 and DHHC7, lower fluorescent signal was also detected with HA-DHHC2, 12, 15, 16 and 2 (top panel). The relative amount of immunoprecipitated PPT1 is shown (middle panel). The expression of the DHHC enzymes in the cell lysates was verified by immunoblotting with anti-HA antibodies (low panel).
(TIF)

## Abbreviations

PPT1: Palmitoyl-Protein Thioesterase 1
NCL: Neuronal Ceroid Lipofuscinosis
DHHC: palmitoylation enzymes with tetrapeptide motif composed of aspartate-histidine-histidine-cysteine (Asp-His-His-Cys)
17 ODYA: 17-octadecynoic acid
BSA: bovine serum albumin
ER: Endoplasmic reticulum
HA: Human influenza hemagglutinin tag
IP: immunoprecipitation
IB: immunoblot
