# Supplementary figures and images for: Reversible Cysteine Acylation Regulates the Activity of Human Palmitoyl-Protein Thioesterase 1 (PPT1)
Source: PLoS One. 2016 Jan 5;11(1):e0146466. doi: 10.1371/journal.pone.0146466 (PMC4701722; doi:10.1371/journal.pone.0146466)

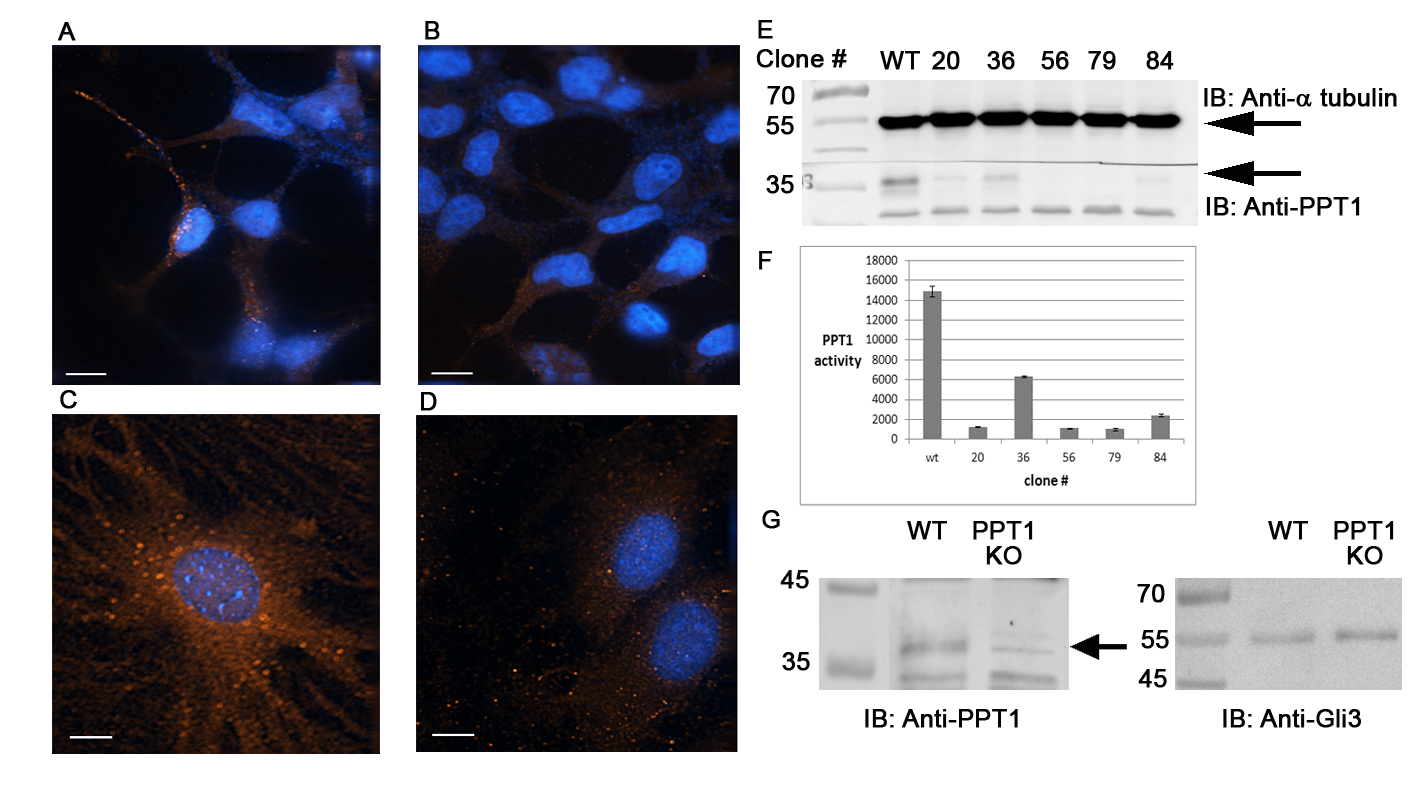

Supplement: S1 Fig — A-B) Overexpression of PPT1 (A) or a control empty plasmid (B) in NIH3T3 cells stained with our rabbit anti-PPT1 antibodies. PPT1 is expressed as discrete puncta with partial localization to perinuclear areas. C-D) Endogenous immuonostaining for mouse PPT1 in MEFs isolated from wildtype mice (C) or PPT1 KO mice (D). PPT1 localizes in various size puncta with preference to perinuclear regions. E-F) Verification of rabbit anti-PPT1 antibodies by western blot. CRISPR/Cas9 gene editing technology was used to generate cell lines deleted for Ppt1. The upper western blot represents similar amount of cell lysates that were loaded using anti α-tubulin antibodies. The lower panel is blotted with anti-PPT1 antibodies. A PPT1 specific band appears in the WT lysates inin close proximity to the 35kDa size marker, which is the expected molecular weight of this protein. Less reactivity isdetected in lysates of clones 36, 20 and 84. No PPT1 immunoreactive proteins were detected in clones 56 and 79. We confirmed those results using an in vitro activity assay for PPT1 activity (F). Cells lysates were incubated with an artificial PPT1 substrate and enzymatic activity was measured. The enzymatic activity correlated with the amounts of protein detected by Western blot. The wildtype lysate exhibited high enzymatic activity, clone 36 less than half of the wildtype activity, lysates of clones 20 and 84 exhibited very low activity, and lysates of clones 56 and 79 displayed no PPT1 activity at all. G) Detection of PPT1 in cilia isolated from cortical cultures. Cilia were isolated as described using high calcium buffer. We could detect PPT1 protein (black arrow) only in the preparation made from the wildtype cortical culture. Cilia enrichment was demonstrated using anti-Gli3 antibodies (no reactivity was seen in the general lysates). (TIF) [file pone.0146466.s001.tif]

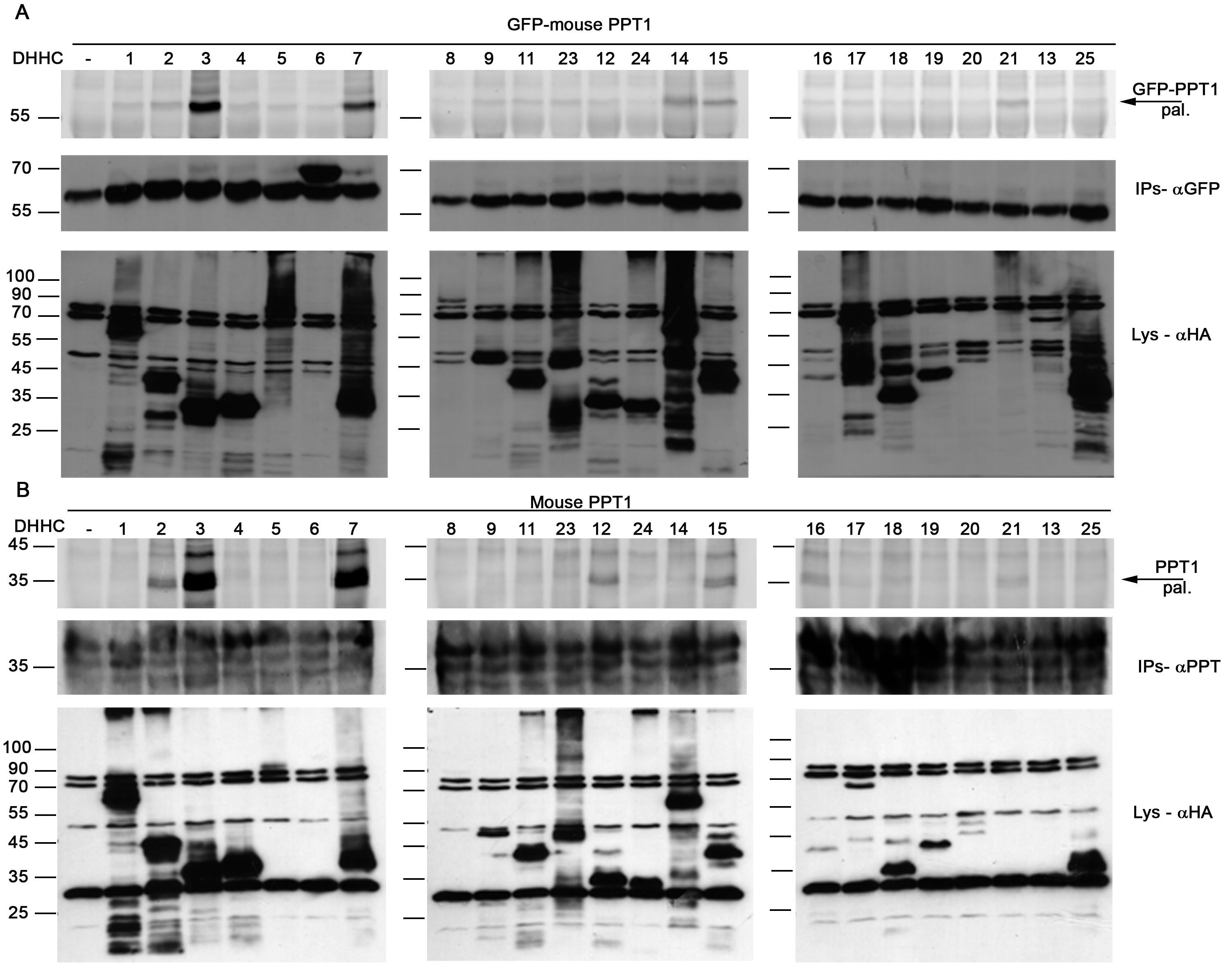

Supplement: S2 Fig — A) GFP-mouse PPT1 is palmitoylated mainly by DHHC3 and DHHC7 palmitoylation enzymes. GFP-mouse PPT1 was co-transfected with HA tagged DHHC enzymes to HEK293 cells. The cells were metabolically labeled with 17-ODYA and GFP-mouse PPT1 was immunoprecipitated with anti-GFP antibodies. A fluorescent tag was introduced by click chemistry and samples were separated by SDS-PAGE. Fluorescent scanning of the gels detected that PPT1 is palmitoylated mainly by DHHD3 and DHHC7 (top panel), a reduced fluorescent signal was detected following the transfections of DHHC14, 15 and 21. The relative amount of immunoprecipitated PPT1 is shown (middle panel). The expression of the DHHC enzymes in the cell lysates was verified by immunoblotting with anti-HA antibodies (low panel). B) Mouse PPT1 is palmitoylated mainly by DHHC3 and DHHC7. A mouse PPT1 expression construct without any added epitope tag was co-transfected with 23 DHHC enzymes to HEK293 cells. The cells were metabolically labeled with 17-ODYA and immunoprecipitated rabbit polyclonal anti-PPT1 antibodies. A fluorescent tag was introduced by click chemistry and samples were separated by SDS-PAGE. Fluorescent scanning of the gels detected that PPT1 is palmitoylated mainly by DHHD3 and DHHC7, lower fluorescent signal was also detected with HA-DHHC2, 12, 15, 16 and 2 (top panel). The relative amount of immunoprecipitated PPT1 is shown (middle panel). The expression of the DHHC enzymes in the cell lysates was verified by immunoblotting with anti-HA antibodies (low panel). (TIF) [file pone.0146466.s002.tif]
